# PW03-012 – Unmet need in Behçet's disease: remission is rare

**DOI:** 10.1186/1546-0096-11-S1-A238

**Published:** 2013-11-08

**Authors:** F Alibaz-Oner, G Mumcu, Z Kubilay, G Ozen, G Celik, A Karadeniz, M Can, S Yılmaz Oner, N Inanc, P Atagündüz, H Direskeneli

**Affiliations:** 1 Rheumatology, Marmara University School of Medicine, Istanbul, Turkey; 2 Health Informatics and Technologies., Marmara University, Faculty of Health Sciences, Istanbul, Turkey; 3 Internal Medicine, Marmara University School Of Medicine, Istanbul, Turkey

## Introduction

The clinical course of Behcet’s disease (BD) as a multi-systemic disorder with a remitting-relapsing nature is unsufficiently explored. As complete remission should be aimed in all inflammatory diseases, we investigated the frequency of complete remission in patients with BD in routine practice.

## Methods

In this retrospective study, 258 patients with BD (F/M: 130/128, mean age: 41.1±11,5 years) classified according to ISG criteria were included. The demographic and clinical data for active organ manifestations and treatment protocols were evaluated, both for the current visit and in the last month. Patients having at least one of any disease manifestations were categorized as active.

## Results

**A t**otal of 1757 visits of 258 patients were overviewed**.** Mean visit number was 6,8± 2,7 (range:1-10) and mean follow-up duration was 45.8±36.5 months (2-165). One hundred twenty-five patients (48.4%) were of mucocutaneus type, whereas 133 patients (51.6%) had major organ involvement. When all visits combined, 19.8-43.9% of the patients were using immunosuppressives (IS), whereas 35.3-59.3% was under non-IS therapies such as colchicine or NSAIDs. There was also a group of noncompliant patients (6.4-45%) without any treatment in some visits. Patients were clinically active in 67.2% (n=1182) of the total visits (n=1757). Frequency of clinical activity increased to 75.6% (68.1- 90.3) when the month before the visit was also included. The major cause of the activity was aphthous ulcers (39.4-63.2%) with other mucocutaneous manifestations also commonly present (Genital ulcer: 3.5-27.1 %, erythema nodosum: 8.2-22.5%, papulopustular lesions: 18.2-33.7%, arthritis: 21.3-33.5%, uveitis: 0.5-8.5% and vascular involvement: 2.5-10.8%). No difference was observed between the frequency of activity of patients having ISs or non-IS therapies.

## Conclusion

Although complete remission is the current, primary target in inflammatory rheumatological diseases such as rheumatoid arthritis or vasculitides, it is fairly difficult to achieve complete remission in BD with current therapeutic regimens. The reluctance of the clinicians to be aggressive for some BD manifestations with low morbidity, such as mucocutaneous lesions, might be influencing the continuous, low-disease activity state in BD patients.

## Disclosure of interest

None declared

